# Penile and preputial pythiosis in Saudi Arabian horses: clinicopathological features and surgical outcomes

**DOI:** 10.3389/fvets.2026.1872270

**Published:** 2026-07-22

**Authors:** El-Sayed El-Shafaey, Mohamed Salem, Walla Awadin, Esam Mosbah, Mohamed Abdo Rizk, Ahmed M. A. Meligy, Saad Shousha, Ahmed Aljazzar, Mahmoud G. El Sebaei

**Affiliations:** 1 Department of Surgery, Anesthesiology and Radiology, Faculty of Veterinary Medicine, Mansoura University, Mansoura, Egypt; 2 Department of Veterinary Surgery, Salam Veterinary Group, Buraydah, Saudi Arabia; 3 Department of Veterinary Clinical Sciences, Faculty of Veterinary Medicine, Jordan University of Science and Technology, Irbid, Jordan; 4 Department of Pathology, Faculty of Veterinary Medicine, Mansoura University, Mansoura, Egypt; 5 Department of Clinical Sciences, College of Veterinary Medicine, King Faisal University, Al-Ahsa, Saudi Arabia; 6 Department of Biomedical Sciences, College of Veterinary Medicine, King Faisal University, Al-Ahsa, Saudi Arabia; 7 Department of Pathology, College of Veterinary Medicine, King Faisal University, Al Ahsa, Saudi Arabia; 8 Department of Biomedical Sciences, College of Clinical Pharmacy, King Faisal University, Al-Ahsa, Saudi Arabia

**Keywords:** clinicopathological, equine pythiosis, penis, prepuce, *Pythium insidiosum*, surgical

## Abstract

**Background:**

Pythiosis, caused by *Pythium insidiosum*, is a chronic granulomatous disease of horses characterized by invasive, ulcerative, and pyogranulomatous cutaneous lesions. Involvement of the external genitalia is uncommon and remains poorly characterized, particularly regarding environmental exposure and treatment outcomes. This study aimed to describe the epidemiological pattern, clinicopathological features, diagnostic findings, and surgical outcomes of penile and preputial genital pythiosis in a hospital-presented horse cohort.

**Methods:**

A retrospective study was conducted on 123 horses presented with cutaneous ulcerative granulomatous lesions to the Horse Hospital, Salam Veterinary Group, between October 2022 and September 2025. Of these, 17 male horses with confirmed penile or preputial involvement were included. Clinical data, lesion characteristics, and outcomes were analyzed. Diagnosis was confirmed by histopathology, immunohistochemistry, and mycological culture, with ultrastructural identification by scanning electron microscopy. All cases were treated by complete surgical excision.

**Results:**

The mean age of affected horses was 6.5 ± 1.8 years, with the majority of cases occurring in gray-colored horses (58.82%). Lesions were predominantly located on the prepuce (52.94%), followed by the urethral orifice (29.41%), glans penis (11.77%), and penile body (5.88). Morphologically, lesions were mainly elliptical (58.8%) or circular (35.3%), with a median surface area of 25.0 cm^2^ (range: 9.0–117.0). Clinically, lesions appeared as ulcerative granulomatous masses with multiple draining tracts and serosanguinous discharge. Histopathological examination revealed eosinophilic granulomas with necrotic centers and peripheral hyphal elements, while immunohistochemistry confirmed *P. insidiosum* infection. Mycological culture demonstrated characteristic growth at 37 °C, and scanning electron microscopy identified characteristic sporangia and oogonia. Surgical excision resulted in complete recovery in 82.35% of cases, while recurrence was observed in 17.65%, particularly in larger surface areas or in multiple preputial lesions.

**Conclusion:**

Early accurate diagnosis and complete excision are essential to minimize recurrence, especially in extensive preputial lesions. This study provides one of the most focused characterizations of genital pythiosis in horses and highlights key prognostic factors relevant to clinical management.

## 1 Introduction

Pythiosis is a chronic granulomatous disease of the skin and subcutaneous tissues caused by *Pythium insidiosum*, an aquatic oomycete that invades host tissues via motile zoospores (1, 2). The disease affects multiple animal species and humans, with horses among the most frequently affected (3–7). In horses, pythiosis typically presents as rapidly progressive ulcerative and pyogranulomatous lesions, commonly involving the distal limbs, ventral abdomen, and thoracic regions. While cutaneous and subcutaneous forms predominate, rarer atypical manifestations are increasingly recognized, including ocular pythiosis causing severe ulcerative keratomycosis (8, 9) and gastrointestinal forms presenting as obstructive segmental enteritis or chronic eosinophilic granulomas (2, 10–13). These lesions are characterized by necrotic masses (kunkers), draining tracts exuding irregularly shaped yellow-tan to grey, gritty, coral-like masses that contain a serosanguinous (yellowish cassatas) material (10). The condition is often associated with intense pruritus and progressive tissue destruction, which can significantly impair animal welfare and performance (11, 12). To ensure accurate clinical recognition, pythiosis must be differentiated from other common proliferative, ulcerative, and granulomatous equine cutaneous lesions (Table 1).

**Table 1:** Comparative differential diagnoses of pythiosis with equine cutaneous habronemiasis, bacterial granulomas (botryomycosis), exuberant granulation tissue (proud flesh), and sarcoids.

Differential diagnosis (10–14, 20)	Clinical features	Gross features	Histopathological features
Pythiosis (*Pythium insidiosum*)	Rapidly progressive, ulcerated, pruritic masses on distal limbs, ventrum, or genitalia.	Pathognomonic kunkers (yellowish-white, coral-like, gritty necrotic cores); serosanguineous discharge.	Eosinophilic to pyogranulomatous inflammation; poorly staining, broad, sparsely septate hyphae visible via GMS or PAS stains.
Habronemiasis (*Habronema*/*Drashia* spp.)	Exuberant granulomas on the medial canthus, prepuce, or wounds; highly pruritic; seasonal (summer).	Small, gritty, yellow sulfur granules calcifications (<2–5 mm); no large branching kunkers.	Severe eosinophilic dermatitis/granulomas containing nematode larvae or larval debris cross-sections.
Bacterial Granuloma (Botryomycosis)	Firm, slow-growing nodules or ulcerated masses, often at surgical sites or pressure points.	Multiple draining tracts discharging purulent exudate with sand-like granules (Splendore-Hoeppli material).	Chronic pyogranulomatous inflammation with central microcolonies of cocci (typically *Staphylococcus aureus*).
Exuberant Granulation Tissue (Proud flesh)	Proliferative, non-healing flesh over existing distal limb wounds; non-pruritic.	Pink to red, cobblestone-like vascular tissue overflowing wound margins; lacks discrete internal necrotic masses.	Dense fibrovascular proliferation, parallel capillary loops, minimal active granulomatous focus unless infected.
Sarcoid (Fibroblastic/Verrucous)	Variable (ulcerated, verrucous, fibroblastic, or nodular masses); single or multiple; non-pruritic.	Fleshy, invasive mass resembling proud flesh but lacking kunkers, sulfur granules, or purulent tracts.	Epidermal hyperplasia with rete peg down-growth, dermal proliferation of atypical fibroblasts in whorls or pickets.

Infection with *Pythium insidiosum* characteristically drives a dominant T helper 2 (Th2) immune response in the host, marked by elevated levels of IL-4, IL-5, and IL-10. This classic polarization promotes a severe eosinophilic and pyogranulomatous inflammatory reaction. Mast cells and eosinophils rapidly recruit and degranulate over the invading oomycete hyphae (6, 7). This massive release of cationic proteins and cellular debris encapsulates the hyphal elements, culminating in a Splendore-Hoeppli-like phenomenon. Especially prominent in equids, this intensive tissue reaction calcifies or hardens into firm, gritty, yellowish-white necrotic concretions known as kunkers, which effectively wall off the viable pathogen from further host immune clearance (11–13).

Despite the relatively well-documented nature of cutaneous pythiosis, involvement of the external genitalia remains rare and poorly characterized in the literature. Genital lesions present specific diagnostic and therapeutic challenges due to anatomical complexity, increased susceptibility to contamination, and difficulty in achieving complete surgical excision (10). Furthermore, the epidemiological and clinical behavior of genital pythiosis, particularly in horses in tropical settings and subjected to mechanical irritation, which plays a critical role in disease transmission, has not been extensively investigated (13).

Diagnosis relies on a combination of clinical findings and laboratory confirmation, including histopathology, immunohistochemistry, and culture. However, early diagnosis remains challenging, and delayed recognition may adversely affect treatment outcomes (3, 11, 14). Therapeutic management of equine pythiosis is often difficult (15, 16). While various medical treatments like immunotherapy utilizing a therapeutic vaccine containing P insidiosum antigens and topical use of antifungal ointment have been proposed, complete surgical excision remains the most effective approach, although recurrence may occur, particularly in extensive or anatomically complex lesions, emphasizing the need for early intervention and precise surgical planning (3, 10).

Given the limited data on genital pythiosis in horses, to the authors’ knowledge, this study represents one of the most comprehensive case series focusing specifically on penile and preputial pythiosis in horses. Thus, the present study aimed to describe the epidemiological factors, clinicopathological features, diagnostic findings, and surgical outcomes of penile and preputial pythiosis in horses, providing insights to improve understanding of the disease and support more effective diagnosis and management strategies.

## 2 Materials and methods

### 2.1 Animals and study design

During the study period between October 2022 and September 2025, 123 horses from Riyadh, Qassim, and Northern provinces, which have a high horse density, were examined at the Equine Hospital, Salam Veterinary Group, for evaluation of cutaneous and/or genital ulcerative granulomatous lesions suggestive of pythiosis. Of these, 17 male horses met the inclusion criteria and were included in the final study cohort. The cohort comprised various skin-colored horses (10 Gray, 5 Chestnut, and 2 Bay), with an age range of 3–9 years (Mean ± SD: 6.5 ± 1.8 years) and a weight range of 350–460 kg (Mean ± SD: 405 ± 45 kg). The Committee of Animal Welfare and Ethics at Salam Veterinary Group, the Horse Hospital, Qassim, Saudi Arabia (SVG.26.04.274), approved the study protocol.

### 2.2 Inclusion and exclusion criteria

Horses were included in this study if they met the following criteria: Presence of ulcerative granulomatous lesions affecting the external genitalia consistent with suspected pythiosis, regardless of size or duration. Definitive diagnosis of pythiosis confirmed by at least one of the following methods: histopathological examination, immunohistochemistry specific for *Pythium insidiosum*, or mycological culture. Availability of follow-up data sufficient to determine treatment outcome (recovery or recurrence). Horses were excluded from the study if they met any of the following conditions: Lesions located outside the external genitalia or a lack of definitive diagnostic confirmation of *Pythium insidiosum*.

### 2.3 Clinical examinations

Upon admission, all horses with suspected genital pythiosis underwent a thorough clinical examination of the lesion. The age, body weight, sex, and color of each horse were recorded in conjunction with the size and location of the lesion. Physical examination of the vital signs and palpation of the superficial regional lymph nodes were conducted.

### 2.4 Hematobiochemical examinations

Upon initial clinical presentation and prior to any surgical intervention, whole blood samples were collected from all affected horses (*n* = 17) via jugular venipuncture. Blood was collected into ethylene diamine tetra acetic acid (EDTA) tubes for haematological analysis and into plain clot-activator tubes for serum biochemical profiling. All samples were processed and analysed at the central diagnostic laboratories of the Salam Equine Hospital (Salam Veterinary Group, Buraydah, Saudi Arabia) within two hours of collection. Complete blood counts (CBC) were performed using an automated veterinary hematology analyser, and differential leukocyte counts were verified manually via Wright-Giemsa stained peripheral blood smears. Serum was separated by centrifugation at 3,000 X g for 10 min; biochemical parameters including liver enzymes, renal markers, total protein, and albumin were measured using an automated clinical chemistry analyser. Plasma fibrinogen concentration was determined via the heat precipitation method.

### 2.5 Mycological examination

Tissue and kunkers samples were rinsed with sterile saline and transported in ampicillin solution (100 mg/mL in sterile saline) to minimize bacterial contamination. Following inoculation, samples were cultured on Sabouraud dextrose agar and incubated at both 25 °C and 37 °C. Plates were examined daily for up to three days for fungal growth. Identification of isolates was confirmed using scanning electron microscopy (17).

### 2.6 Histopathological and immunohistochemical examinations

Representative tissue samples were collected from the lesions and fixed in 10% neutral-buffered formalin. Following routine processing, paraffin-embedded sections (5 μm thick) were stained with hematoxylin and eosin (H&E), Masson trichrome (MT), and Grocott’s Methenamine Silver GMS for histopathological evaluation of the host tissue damage and scarring. For immunohistochemical confirmation, selected sections were stained using a labeled streptavidin–biotin peroxidase method. A horse-derived polyclonal antibody against *Pythium insidiosum* was applied (3).

### 2.7 Surgical treatment

Before surgery, horses underwent a complete hematological and biochemical profiling to ensure the absence of anemia or organ failure, and to allow the patient to undergo surgery. Following a 12-h fast, horses received preoperative cefotaxim (3 mg/kg, IM) and Flunixin meglumine (1.1 mg/kg, IV). The animals were regionally or generally anesthetized according to the location of the swelling. General anesthesia was obtained using an infusion regimen of xylazine (1.1 mg/kg, IV) and Ketamine HCl (2.2 mg/kg, IV). While epidural anesthesia was achieved by injection of lidocaine HCl (0.2 mg/ kg) in the 1st intercoccygeal space. After aseptic preparation of the surgical site, a catheter was placed in the urethra and advanced into the bladder as a guide to protect the urethra and to evacuate urine at the end of the procedure. Masses were surgically treated according to their size and site using local excision and/or segmental posthectomy (10), including a margin of apparently healthy tissue to ensure total removal of affected areas. The surrounding skin was undermined to facilitate closure, and wounds were closed using relaxing sutures with No. 1 Polygalactin 910 suture. The preoperative antibiotics and anti-inflammatory medications were continued for five successive days postoperatively. Animals were confined in a stall to rest for 1 week with the wound dressed and monitored daily until suture removal 10 days postoperatively. All operated horses were followed up for about 6 months postoperatively to assess any surgical complications and detect any evidence of recurrence.

### 2.8 Statistical analysis

Data were analyzed using IBM SPSS Statistics (version 24.0). Descriptive statistics were calculated for all variables. Continuous clinical data (age, body weight, and lesion surface area) were assessed for normality using the Shapiro–Wilk test. Because these clinical data were non-parametric, continuous variables are presented as medians and interquartile ranges (IQRs), while categorical variables (color, lesion site, and shape) are presented as absolute frequencies and percentages. In contrast, hematobiochemical data demonstrated a normal distribution and are expressed as mean ± standard deviation (SD). One-sample t-tests were utilized to compare all hematobiochemical values against standard healthy equine reference intervals. Statistical significance was set at *p < 0.05* for all analyses.

## 3 Results

### 3.1 Epidemiological and clinical findings

A total of 17 cases of equine pythiosis were evaluated. The mean age of affected horses was 6.5 ± 1.8 years, with the majority of cases occurring in gray-colored horses (58.82%; *n* = 10). The most frequent anatomical site for lesions was the prepuce (52.94%; *n* = 9), followed by the urethral orifice (29.41%; *n* = 5), glans penis (11.77%; *n* = 2), and penile body (5.88%; *n* = 1). Lesions were predominantly elliptical (58.82%; *n* = 10) or circular (35.30%; *n* = 6) in shape, with a median surface area of 25.0 [9.0–117.0]. Larger lesions were more frequently observed in the preputial region. Descriptive details of the lesions were reported in Table 2 and Figures 1, 2.

**Table 2:** Demographic and clinical characteristics of penile and preputial pythiosis in horse cohort (*n* = 17).

Variable	Level	Frequency (*n* = 17)	%	Mean ± SD	Median (IQR)
Age (years)	-	-	-	6.5 ± 1.8	-
Body weight (kg)	-	-	-	405 ± 45	-
Lesion surface area (cm2)	-	-	-	-	25.0 [9.0–117.0]
Season	Winter	3	17.65%	-	-
	Spring	11	64.70%	-	-
	Summer	1	5.88%	-	-
	Autumn	2	11.77%	-	-
Color	Gray	10	58.82%	-	-
	Chestnut	5	29.41%	-	-
	Bay	2	11.77%	-	-
Anatomical site	prepuce	9	52.94%	-	-
	Urethral orifice	5	29.41%	-	-
	Glans penis	2	11.77%	-	-
	Penile body	1	5.88%	-	-
Lesion shape	Elliptical	10	58.82%	-	-
	Circular	6	35.30%	-	-
	Cylindrical	1	5.88%	-	-
Surgical outcome	Recovered	14	82.35%	-	-
	Recurrent	3	17.65%	-	-

**Figure 1:**
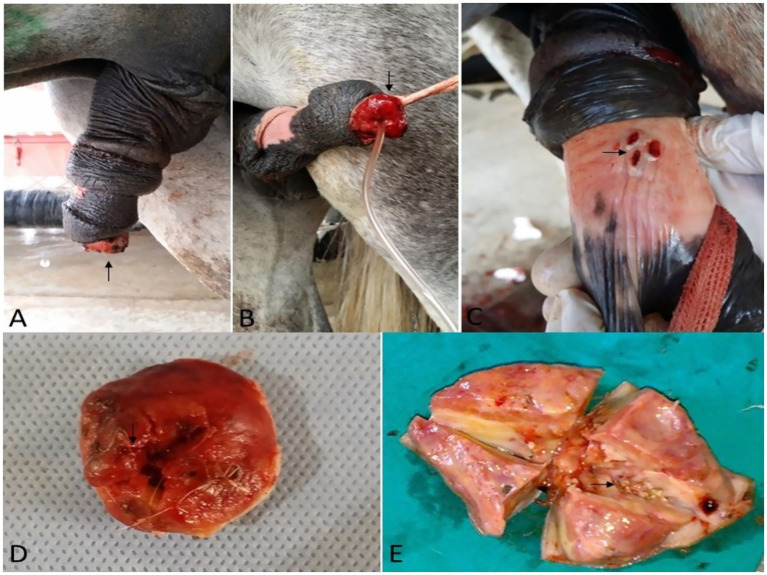
Gross features of penile pythiosis in horses. Gross morphology of ulcerative granulomatous mass on the glans penis **(A)**, urethral orifice **(B)**, and on the penile body **(C)**. **(D,E)** Cut surfaces of the surgically removed penile granulomatous mass revealed characteristic yellow-grey, coral-like materials (kunkers or leeches) within the sinus tracts and the granulomatous tissue (arrow).

**Figure 2:**
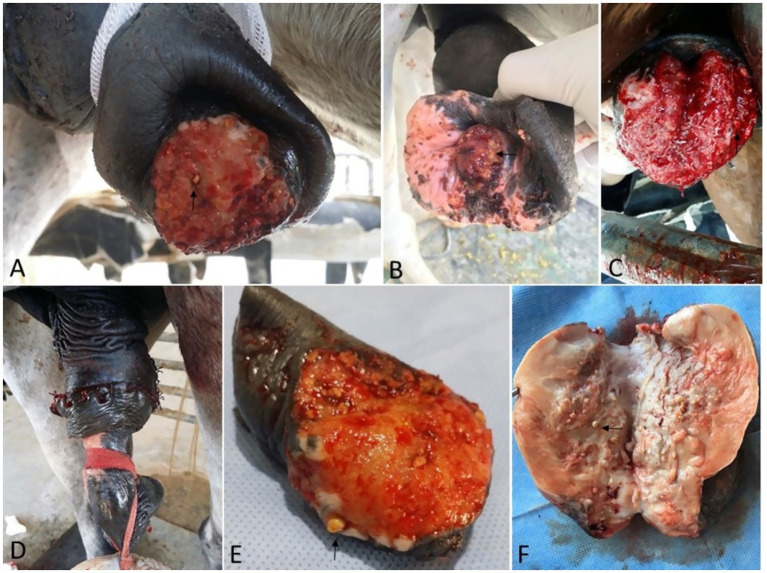
Gross features of preputial pythiosis in horses. Gross morphology of different forms of ulcerative granulomatous mass on the prepuce **(A–C)**, and their surgical treatment with segmental posthectomy **(D)**. **(E,F)** Cut surfaces of the surgically removed preputial granulomatous mass revealed characteristic yellow-grey, coral-like materials (kunkers or leeches) within the sinus tracts and the granulomatous tissue (arrow).

Although cases were reported throughout the year, a higher frequency was observed during the spring season (64.70%; *n* = 11) between March and Jun, suggesting a temporal association between environmental and occupational exposure and the occurrence of genital pythiosis.

Clinically, lesions appeared as ulcerative granulomatous masses composed of whitish-yellow tissue with multiple draining sinus tracts exuding serosanguinous or bloody discharge. Lesions initially developed as small cutaneous nodules and progressively enlarged into alopecic, ulcerated masses associated with marked pruritus. Compression of the lesions resulted in the extrusion of serosanguinous material through multiple sinus openings.

### 3.2 Hematobiochemical findings

Hematological and biochemical evaluation of the 17 affected horses at initial presentation revealed a consistent pattern of systemic chronic inflammation and immune activation (Table 3). A mild-to-moderate normocytic, normochromic anemia was present, characterized by decreased mean red blood cell count (6.20 ± 0.73 ×106/μL), hemoglobin (9.52 ± 0.67 g/dL), and hematocrit (31.07 ± 2.80%). Leukocyte profiles were marked by mature neutrophilia (10,015 ± 159.35/μL) and profound peripheral eosinophilia (2,004 ± 25.71/μL), which stood at double the upper reference limit. Biochemical analyses demonstrated hyperfibrinogenemia (5.03 ± 0.76 g/L) and a shifted protein profile with a low mean albumin-to-globulin (A/G) ratio (0.80). Renal (BUN, creatinine) and hepatic (AST, ALP) parameters remained within standard equine reference ranges.

**Table 3:** Hematological and biochemical findings in 17 horses with penile and preputial pythiosis.

Parameters	Mean ± SD	Reference range
Hematology		
Red blood cells (*RBCs,* x10^6^/μL)	6.20 ± 0.73	8.5–12.5
Hemoglobin (*Hgb,* g/dL)	9.52 ± 0.67	12.0–17.0
Hematocrit (*HCT,* %)	31.07 ± 2.80	33–55
Mean cell volume (*MCV,* f/L)	44.9 ± 1.75	37–55
Mean cell hemoglobin (*MCH,* pg)	15.01 ± 0.73	12.5–20
Mean corpuscular hemoglobin concentration (*MCHC,* g/dL)	33.04 ± 0.51	31.0–38.0
White blood cells (*WBCs,* n/μL)	13,260 ± 1,492.11	6,500–15,000
Lymphocytes (/μL)	2,527 ± 413.78	1,500–6,500
Neutrophils (/μL)	10,015 ± 159.35	2,250–8,500
Monocytes (/μL)	521 ± 29.58	0–1,000
Eosinophils (/μL)	2,004 ± 25.71	0–1,000
Biochemistry		
Fibrinogen (g/L)	5.03 ± 0.76	1–4
Total protein (*TP,* g/L)	64.09 ± 1.92	57–77
Albumin (g/L)	28.56 ± 1.34	25–42
Aspartate aminotransferase (*AST,* UI/L)	203 ± 14.85	155–350
alkaline phosphatase (*ALP,* UI/L)	341.52 ± 17.33	142–400
Blood urea nitrogen (*BUN,* mmol/L)	12.68 ± 0.49	10.0–20.45
Creatinine (*CR,* μmol/L)	115.71 ± 8.93	78.0–160.0

### 3.3 Treatment outcomes

Early and complete surgical excision remains the cornerstone of treatment. Complete surgical excision was performed in all cases. The overall recovery rate was 82.35% (14/17), while recurrence was observed in 17.65% (3/17) of cases, localized to the prepuce and were associated with relatively large surface areas or multiple masses (Table 2).

### 3.4 Mycological findings

Mycological culture at 37 °C yielded white to off-white colonies, whereas no growth was observed at 25 °C (Figure 3A). The colonies lacked aerial mycelium and demonstrated submerged hyphal growth within the medium in every culture. Scanning electron microscopy revealed characteristic features of *Pythium* species, including hyphal swelling with globose sporangia (approximately 21.5 μm in diameter), as well as sexual structures such as globose oogonia (approximately 17 μm in diameter) and diclinous antheridia, which are typical of *Pythium* species (Figure 3B).

**Figure 3:**
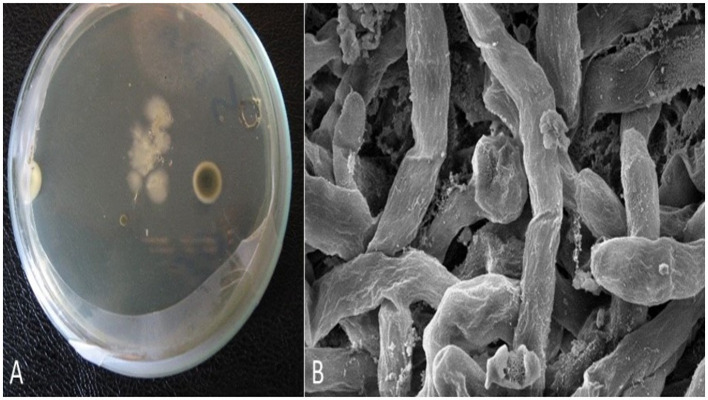
Mycological examination of tissue and kunkers samples cultured on Sabouraud dextrose agar. **(A)** Mycological findings revealed white to off-white colonies. **(B)** Scanning electron microscopy of isolates revealed hyphal swelling with globose sporangia 21.5 mm in diameter and the presence of sexual structure (globose oogonia 17 mm in diameter and diclinous anthredia that are characteristic of Pythium species).

### 3.5 Histopathological and immunohistochemical findings

Histopathological examination demonstrated typical granulomatous inflammation characterized by central eosinophilic necrotic debris surrounded by fibrocollagenous granulation tissue and palisading inflammatory cells. The necrotic core exhibited fissures, particularly at its periphery, and was infiltrated by numerous inflammatory cells, and the presence of kunkers material (Figures 4A,B). Massive fibrocollagenous connective tissue stained blue in the wall of the granuloma by MT stain (Figure 4C). Fungal hyphae were observed predominantly at the margins of the necrotic areas, appearing as negatively stained tubular structures within the tissue sections. In some areas, hyphae were highlighted as dense, branching elements consistent with *Pythium insidiosum* infection (Figure 5A). Immunohistochemical analysis showed strong positive staining of the hyphal walls in lesion samples using anti-*P. insidiosum* antibodies, whereas negative control sections showed no immunoreactivity (Figure 5B).

**Figure 4:**
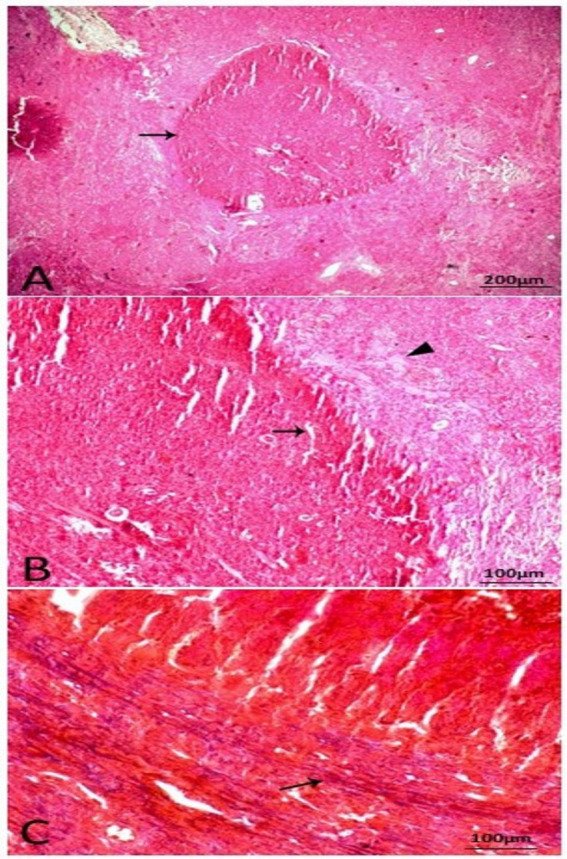
Histomicrograph of penile and preputial pythiosis. **(A)** Cross-section of lesion showing caseated granuloma and fibrocollagenous proliferation in the wall of the granuloma (HE, 40x bar 200). **(B)** Granuloma with caseous necrosis, multiple cracks, especially at the periphery of eosinophilic necrotic tissue (arrow), surrounded by palisade-like cells in subcutaneous tissue (arrowhead) in the skin of an animal infected with *P insidiosum* (HE, 100x bar 100). **(C)** Massive fibrocollagenous connective tissue stained blue (arrow) in the wall of the granuloma (MT, 100x).

**Figure 5:**
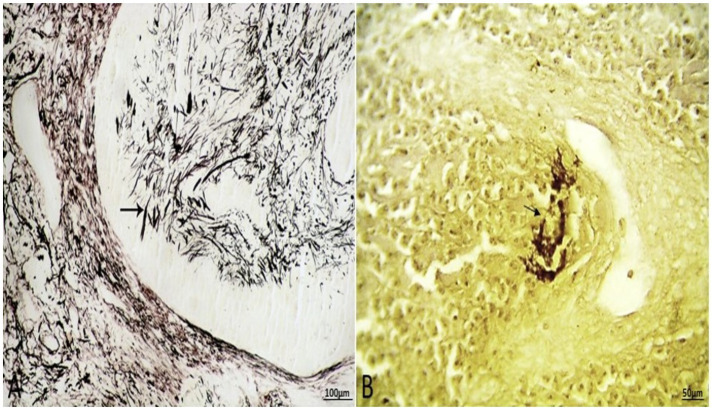
Histomicrograph of penile and preputial pythiosis. **(A)** Fungal hyphae are present in large numbers, stained black (arrow), particularly at the periphery of the granuloma center, indicating that the unstained empty area is thick fungal hyphae (GMS, 100x). **(B)** Walls of the hyphae-like structures stained positively for antibodies against *P. insidiosum* in lesion samples (arrow) (IHC, 400x bar 50).

## 4 Discussion

The present study provides a focused description of penile and preputial pythiosis in horses, highlighting its epidemiological pattern, clinicopathological features, and surgical outcomes. Although equine pythiosis is typically reported as a cutaneous disease affecting distal limbs and ventral body regions, involvement of the external genitalia remains relatively underreported, making the current findings clinically relevant (3, 8, 10). To the authors’ knowledge, this is one of the case series studies specifically describing penile and preputial pythiosis in horses.

The seasonal prevalence of equine penile and preputial pythiosis in this study, particularly between March and June (64.70%; *n* = 11) during the spring season, suggests a strong link between environmental conditions and occupational risk. The convergence of heavy spring rainfall, warm ambient temperatures (25–35 °C), and stagnant water creates an ideal niche for *Pythium insidiosum* zoosporogenesis, particularly in regions such as the prepuce and penis that are prone to trauma and moisture retention (3, 13). We propose that horses are infected when biflagellate zoospores penetrate cutaneous abrasions, likely sustained during spring festivals or grazing upon contact with contaminated water or damp soil. Furthermore, the presence of *Pythium* species in local crop rhizospheres indicates that agricultural runoff and irrigated pastures serve as critical environmental reservoirs (18, 19). These findings highlight that pythiosis in the Arabian breed is an environmentally driven occupational disease, necessitating improved pasture management and preventive strategies in high-risk populations.

The Prepuce accounted for 52.94% of all cases (10/17) and of the observed recurrences. The predominance of preputial lesions (58.3%) could be attributed to the anatomical conformation of the prepuce, which favors prolonged moisture retention, mechanical irritation, and the preputial folds may harbour pathogens more effectively, thereby enhancing susceptibility to infection or present challenges for local treatment (10, 12).

Elliptical lesions were the most common shape (58.82%; *n* = 10), followed by Circular (35.30%; *n* = 6). The median surface area was significantly high, 25.0 [9.0–117.0], indicating that cases are often presented at a mature stage. The variability in lesion morphology and size reflects different stages of disease progression, with larger lesions likely representing chronic cases (3, 20). A higher prevalence was noted in gray horses (58.82%; *n* = 10), which aligns with certain literature suggesting a potential predisposition or higher visibility of lesions in lighter-pigmented horses (11, 21).

Clinically, the lesions observed in this study were consistent with classical descriptions of pythiosis, characterized by ulcerative granulomatous whitish-yellow masses with multiple draining tracts and bloody or serosanguinous exudate (20, 22). The presence of intense pruritus and the extrusion of necrotic material upon compression are hallmark features that can aid in clinical suspicion.

The systemic hematological profiles observed in our cohort provide critical insight into the systemic pathology of equine pythiosis. The normocytic, normochromic anemia detected in these horses is a classic manifestation of the anemia of chronic disease, driven by inflammatory cytokine-mediated suppression of erythropoiesis and altered iron sequestration within the mononuclear phagocyte system. This chronic inflammatory state is further supported by the elevated plasma fibrinogen concentrations. Most notably, the profound peripheral eosinophilia (2,004 ± 25.71/μL) reflects the characteristic, intense T-helper 2 (Th2) polarized immune response elicited by Pythium insidiosum antigens, which fail to clear the pathogen but drive extensive tissue eosinophil recruitment and subsequent fibrogranulomatous remodeling. While none of these hematological anomalies are diagnostic on their own, their collective presentation alongside proliferative genital lesions acts as a strong clinical indicator for pythiosis in endemic regions (7, 11).

Surgical excision resulted in a high success rate (82.35%), which is consistent with previous reports emphasizing the effectiveness of early and complete removal of infected tissue (1–3, 9, 10). The favorable outcomes observed in most cases may be attributed to meticulous surgical technique, including excision with safety margins. However, recurrence occurred in three cases (17.65%), which were localized to the prepuce and were associated with relatively large surface areas or multiple distributions. This finding suggests that lesion extent and anatomical location may influence treatment outcome. The association between larger lesion size and recurrence has been previously suggested and may reflect deeper tissue invasion or incomplete removal of infected margins (2, 23). Furthermore, the complex anatomy of the prepuce may hinder adequate surgical access, increasing the likelihood of residual infection. These findings underscore the importance of early diagnosis and intervention to improve therapeutic outcomes.

The present study provides valuable insights into the clinical behavior of genital pythiosis in horses. The integration of clinical, pathological, and microbiological findings strengthens the diagnostic approach and highlights the importance of combining multiple modalities for accurate identification (11). Histologically, the identification of eosinophilic granulomas with central necrosis, kunkers, and peripheral hyphal elements is characteristic of pythiosis. The negative staining of hyphae in routine sections, along with their localization at the margins of necrotic tissue, is consistent with the known histomorphological features of *P. insidiosum* (12). Immunohistochemical confirmation further strengthened the diagnosis, demonstrating specific antigenic labeling of hyphal walls for antibodies against *P. insidiosum* in lesion samples. Additionally, the growth of colonies at 37 °C but not at 25 °C, along with ultrastructural features of hyphal swelling with globose sporangia 21.5 mm in diameter and the presence of a sexual structure, observed by scanning electron microscopy, supported the differential diagnosis of the organism (6, 7, 14).

This study is limited by the small sample size, retrospective design, and single-center data source, which may restrict statistical power and generalizability. Although diagnosis was confirmed using histopathology, immunohistochemistry, and culture, the absence of molecular techniques such as PCR may limit pathogen characterization. Additionally, the exclusive use of surgical treatment without comparison to other modalities precludes evaluation of relative therapeutic efficacy. Despite these limitations, the findings provide valuable insight into a relatively underreported form of equine pythiosis. Future studies should include larger, multicenter cohorts with prospective designs, standardized protocols, and incorporation of molecular diagnostics. Comparative evaluation of different treatment approaches, as well as further investigation into environmental risk factors and preventive strategies, is warranted to improve disease management and outcomes.

## 5 Conclusion

Genital pythiosis in horses is strongly associated with environmental exposure and mechanical irritation to the equine penis and prepuce. The disease is characterized by distinctive clinicopathological features and can be effectively managed through complete surgical excision. However, lesion size and location, especially in the prepuce, may influence the risk of recurrence. Early recognition and prompt surgical intervention remain critical for achieving favorable outcomes.

## Data Availability

The original contributions presented in the study are included in the article/supplementary material, further inquiries can be directed to the corresponding authors.
